# A combined functional and structural genomics approach identified an EST-SSR marker with complete linkage to the Ligon lintless-2 genetic locus in cotton (*Gossypium hirsutum *L.)

**DOI:** 10.1186/1471-2164-12-445

**Published:** 2011-09-09

**Authors:** Doug J Hinchliffe, Rickie B Turley, Marina Naoumkina, Hee Jin Kim, Yuhong Tang, Kathleen M Yeater, Ping Li, David D Fang

**Affiliations:** 1 USDA-ARS, Southern Regional Research Center, Cotton Fiber Bioscience Research Unit, New Orleans, LA, 70124, USA; 2 USDA-ARS, Southern Regional Research Center, Cotton Chemistry & Utilization Research Unit, New Orleans, LA, 70124, USA; 3 USDA-ARS, Mid South Area, Crop Genetics Research Unit, Stoneville, MS, 38772, USA; 4 The Samuel Roberts Noble Foundation, Genomics Core Facility, Ardmore, OK, 73401, USA; 5 USDA-ARS, Southern Plains Area, College Station, TX, 77845, USA

## Abstract

**Background:**

Cotton fiber length is an important quality attribute to the textile industry and longer fibers can be more efficiently spun into yarns to produce superior fabrics. There is typically a negative correlation between yield and fiber quality traits such as length. An understanding of the regulatory mechanisms controlling fiber length can potentially provide a valuable tool for cotton breeders to improve fiber length while maintaining high yields. The cotton (*Gossypium hirsutum *L.) fiber mutation Ligon lintless-2 is controlled by a single dominant gene (*Li_2_*) that results in significantly shorter fibers than a wild-type. In a near-isogenic state with a wild-type cotton line, *Li_2 _*is a model system with which to study fiber elongation.

**Results:**

Two near-isogenic lines of Ligon lintless-2 (*Li_2_*) cotton, one mutant and one wild-type, were developed through five generations of backcrosses (BC_5_). An F_2 _population was developed from a cross between the two *Li_2 _*near-isogenic lines and used to develop a linkage map of the *Li_2 _*locus on chromosome 18. Five simple sequence repeat (SSR) markers were closely mapped around the *Li_2 _*locus region with two of the markers flanking the *Li_2 _*locus at 0.87 and 0.52 centimorgan. No apparent differences in fiber initiation and early fiber elongation were observed between the mutant ovules and the wild-type ones. Gene expression profiling using microarrays suggested roles of reactive oxygen species (ROS) homeostasis and cytokinin regulation in the *Li_2 _*mutant phenotype. Microarray gene expression data led to successful identification of an EST-SSR marker (NAU3991) that displayed complete linkage to the *Li_2 _*locus.

**Conclusions:**

In the field of cotton genomics, we report the first successful conversion of gene expression data into an SSR marker that is associated with a genomic region harboring a gene responsible for a fiber trait. The EST-derived SSR marker NAU3991 displayed complete linkage to the *Li_2 _*locus on chromosome 18 and resided in a gene with similarity to a putative plectin-related protein. The complete linkage suggests that this expressed sequence may be the *Li_2 _*gene.

## Background

Cotton seed fibers are initially ovule epidermal cells that terminally differentiate into fiber cells typically on the DOA. Approximately 25% of the ovule epidermal cells differentiate into fiber cells during the initiation stage of cotton fiber development and subsequently undergo a period of rapid elongation known as the elongation stage [1,2]. The rate of fiber elongation peaks at approximately 6 to 12 DPA and nears cessation at 22 DPA [3]. During peak elongation fiber cells can increase in length at rates of 2 mm/day or more depending on environment and genotype [1,4]. The length of fibers is mostly variety specific, but can also be affected by environmental conditions such as temperature during the elongation stage of development [5]. The elongation stage is followed by a brief period known as the transition stage that usually begins from 12 to 16 DPA in field conditions and depending on environmental factors such as lower temperatures that are shown to delay the onset of the transition stage [6]. The SCW stage immediately follows transition and is characterized by a dramatic increase in SCW-related gene transcripts like cellulose synthases and changes in cell wall composition as large amounts of cellulose are deposited in the SCW [7]. The SCW stage persists until about 32 DPA at which time the fiber cell is composed of approximately 95% cellulose with the remaining 5% of non-cellulosic materials comprised of proteins, polysaccharides, pectins, and waxes that reside mostly in the PCW and cuticle [3,8]. The final stage of fiber development is maturation that ceases from 40 to 60 DPA depending on environment and genotype [9]. At this time the cotton bolls crack and open, exposing the seed fibers to external ambient conditions causing them to desiccate and take on the fluffy appearance normally associated with cotton fibers.

Varieties of cultivated Upland cotton (*Gossypium hirsutum *L.) that display fiber mutation phenotypes including lintless and fuzzless seeds were first described in the early twentieth century [10,11]. Currently, numerous naturally occurring cotton fiber mutations have been identified globally and characterized at the genetic, and more recently, gene expression levels [12-16]. These fiber mutations include, among others, the glabrous seeds in the fiberless mutant lines MD17, SL1-7-1, and XZ142w [17,18]; seeds with only lint fibers and no fuzz fibers in the Naked seed lines *N_1 _*[11] and *n_2 _*[19]; and seeds that are described as extremely short lint fibers in the Ligon lintless-1 (*Li_1_*) [20] and Ligon lintless-2 (*Li_2_*) [21] mutant lines. The fiber mutations of *N_1_*, *Li_1_*, and *Li_2 _*are single gene dominant traits [20,21] while the *n_2 _*fiber mutation is a single gene recessive trait [19]. Recent genetic studies on the *Li_2 _*mutation also indicate that it may have incomplete penetrance as evidenced by mutant and WT fibers at different boll locations on the same plants [22], or possibly phenotype variation due to epigenetics. These naturally occurring mutants and their wild-type fiber NILs provide a unique and powerful model system to study cotton fibers at various stages of development including initiation, elongation, and secondary cell wall biosynthesis.

The *Li_1 _*gene was mapped to chromosome 22 using SSR [23] and RFLP [24] markers while the *Li_2 _*gene was mapped to chromosome 18 by phenotype association with cotton aneuploid stocks [25], and linkage analysis by RFLP markers [24]. More recently, a draft of the physical map of the diploid cotton D-genome progenitor *G. raimondii *was released and used along with tetraploid cotton A- and D- subgenome genetic maps to generate a consensus genetic-physical map of the cotton genome that included flanking markers for the *Li_2 _*gene on chromosome 18 [26]. These were RFLP markers designated A1552 and Gate4BC11 that flanked the *Li_2 _*gene in an 8.9 cM region and were mapped using an interspecific F_2 _mapping population composed of 158 individuals from a cross between *G. hirsutum Li_2 _*and *G. barbadense *cv. Pima S-7. Previously, another RFLP marker designated Gate4BF10 was reported to flank the *Li_2 _*gene along with A1552 in a 1.5 cM region of chromosome 18 [24]. However, the more recently released cotton consensus genetic-physical map developed by the same laboratory indicated that Gate4BF10 was mapped at two locations on chromosome 18 [26], which leave the mapping accuracy of this marker in doubt. In a separate study, two *Li_2 _*F_2 _segregating populations were developed and used to screen SSR markers for linkage to the *Li_2 _*genetic locus. The closest SSR marker was mapped to chromosome 18 and located 6.051 cM from the *Li_2 _*gene in an interspecific segregating population, and 9.266 cM from the *Li_2 _*gene in an intraspecific segregating population [27].

In a near-isogenic state with the cotton line Texas Marker-1 (TM-1), both the *Li_1 _*and *Li_2 _*mutants have seed fibers that are extremely short (< 6 mm) compared to WT fibers that are typically greater than 20 mm in length [20,21,28]. As a monogenic dominant trait, the short-fiber phenotypes of *Li_1 _*and *Li_2 _*are identical in either a homozygous dominant or heterozygous state. Unlike the *Li_1 _*mutant, which exhibits pleiotropy in the form of severely stunted and deformed plants in both the homozygous dominant and heterozygous state [20], the *Li_2 _*mutant plants appear healthy and morphologically identical to the homozygous recessive wild-type plants with the exception of shorter seed fibers [21].

Cytological evidence suggests that the seed fibers of *Li_1 _*mutants undergo initiation in the same manner as WT fibers, but begin to show some distorted morphological features during the early elongation stage of development [23]. Since the seed fibers of *Li_1 _*and *Li_2 _*fibers are shortened lint fibers, these cotton mutants represent excellent candidates to study the molecular mechanisms of fiber elongation. A recent gene expression study using microarrays on *Li_1 _*mutant and WT cotton NILs that focused on the SCW stage of fiber development identified genes potentially responsible for the phenotypic differences observed in mutant *Li_1 _*fibers compared to WT fibers [12]. Several genes in particular were differentially expressed during SCW biosynthesis that could potentially be involved with the *Li_1 _*phenotype including *EXPANSINS*, tubulin genes, sucrose synthase (*SuSy*), and genes encoding MYB transcription factors [12].

Since extensive research has been ongoing with the *Li_1 _*mutant, our laboratory selected the *Li_2 _*mutant in an established near-isogenic state with the Upland cotton variety DP5690 as a model system to study fiber elongation events using a combined functional and structural genomics approach of microarray gene expression and molecular marker analysis. The *Li_2 _*mutant was also selected due to concerns over the pleiotropic effects of the *Li_1 _*mutation on development timing and the desire to harvest fiber samples from mutant and WT plants simultaneously. Understanding the molecular events that control fiber elongation and identifying regulatory elements involved in this process can provide cotton researchers and breeders with means of improving fiber length while maintaining yield either through marker-assisted selection or a transgenic approach. The main objective of this research was to identify genes that were differentially expressed during the development of WT and mutant *Li_2 _*fibers and convert the gene expression data into portable molecular markers for use in association mapping to identify the *Li_2 _*locus.

Here we report: 1) the development of two *Li_2 _*NILs of cotton (*G. hirsutum*) in the backcross five (BC_5_) generation; 2) no apparent phenotypic differences in seed fibers of mutant *Li_2_Li_2 _*plants compared to WT *li_2_li_2 _*plants during the initiation and early elongation stages of fiber development; 3) mapping the *Li_2 _*locus with SSR markers; 4) the identification of genes differentially expressed in fibers of mutant *Li_2 _*plants compared to WT plants using microarray gene expression analysis on selected developmental time-points; 5) confirmation of microarray gene expression profiles by RT-qPCR; 6) successful conversion of differentially expressed genes into EST-SSR markers; and 7) the identification of an EST-SSR marker representing a putative plectin-related protein or regulatory element that has complete linkage to the *Li_2 _*genetic locus suggesting it may be the *Li_2 _*gene.

## Methods

### Plant materials and greenhouse experimental design

Two NILs of *Li_2 _*Upland cottons that were homozygous dominant (*Li_2_Li_2_*) and homozygous recessive (*li_2_li_2_*) for the *Li_2 _*locus were developed in a backcross program at Stoneville, MS in field and greenhouse environments. Mutant Texas marker-1 (TM-1) cotton plants containing the *Li_2 _*gene were crossed with the Upland cotton variety DP5690 and F_1 _progeny were backcrossed for five generations (BC_5_) by SSD to DP5690 which served as the recurrent parent in each backcross. The DP5690 recurrent parent was a pure inbred line that was self-pollinated for nine generations via SSD. Progeny in each backcross were selected based on phenotype for the *Li_2 _*short-fiber mutation. The pedigree of the two *Li_2 _*NILs is detailed in Additional file 1.

A total of 102 *Li_2 _*cotton plants and 80 WT *li_2_li_2 _*cotton plants were planted in the greenhouse on six tables. The mutant *Li_2 _*plants were six BC_5_F_3 _lines that originated via SSD. A total of 17 individual plants were grown for each line to confirm that they were homozygous *Li_2_Li_2 _*[29]. Once the genotypes were confirmed the populations were culled to 72 mutant *Li_2_Li_2 _*plants and 72 WT *li_2_li_2 _*plants that were placed in the same greenhouse on six tables in a randomized complete block design. The individual mutant and WT cotton plants were labeled into three pools representing three biological replicates. Cotton bolls were harvested at the following time-points during development: -3, -1, 0, 1, 3, 5, 8, 12, 16, and 20 DPA. Bolls from the same cotton line, biological replicate, and developmental time-point were harvested from all six tables to account for environmental variability within the greenhouse and bulked for subsequent analyses. The number of bolls per bulked sample varied according to developmental time-point, with a greater number of bolls required for the earliest time-point to ensure sufficient biological material and progressively fewer bolls required for each successive time-point. For example, at opposite ends of the developmental time-course, ovules from approximately 20 - 30 bolls were bulked for each -3 DPA sample, and ovules with fibers attached from approximately 8 -10 bolls were bulked for each 20 DPA sample. Harvested bolls were placed immediately on ice and transported to the laboratory where they were dissected on ice and the majority of the ovules frozen in liquid nitrogen and stored at -80°C. A small number of ovules from each sample from the -1 to 5 DPA time-points were used for SEM as described herein.

### Mapping population

A mutant *Li_2_Li_2 _*homozygous plant was used as the female in a cross with its near-isoline WT *li_2_li_2 _*DP5690. One hundred and thirty-six F_2 _plants were planted in the field in Stoneville, MS in 2009. The *Li_2 _*trait of each F_2 _progeny plant was evaluated twice at approximately 30 DPA and after boll maturation and opening.

### SSR marker analysis and genetic mapping

Young leaves were collected from each one of the F_2 _plants in the described mapping population. Total DNA was extracted from fresh leaves using 2.0% hexadecyltrimethylammonium bromide [30]. DNA was purified using Omega EZNA^® ^DNA isolation column (Omega Bio-Tek, Norcross, GA). As previously reported, the *Li_2 _*locus resides on chromosome 18 [24,25,27]. To rapidly identify SSR markers closely linked to the *Li_2 _*locus, we first selected 86 SSR markers that were previously mapped on either chromosome 18 or its homeologous chromosome 13 based on several published maps [31-35]. The probes of the RFLP markers reported by Rong et al. (2005) [24] were not available to us, and thus were not evaluated in our population. Bulked segregant analysis [36] was then used to identify potentially linked markers. For the *Li_2 _*bulk, DNA of 10 F_2 _plants that had the *Li_2 _*phenotype were pooled at equal ratio and diluted to 10 ng/μL. The WT bulk consisted of pooled DNA from 10 F_2 _progeny that had normal lint phenotype. SSR primers that generated polymorphic patterns between bulks were tested using the 20 individual DNA samples that were included in the bulks. The markers linked to the *Li_2 _*locus were analyzed on 136 individual F_2 _progeny plants as previously described [37]. All SSR primer sequences can be obtained from Cotton Marker database (http://www.cottonmarker.org) except DPL0547. The primer sequences of the SSR markers associated with *Li_2 _*locus are listed in Additional file 2. Segregation data for the *Li_2 _*trait and SSR markers were mapped using program JoinMap3.0 [38,39] with logarithm of odds score = 25.

### Scanning electron microscopy

To prepare the samples for SEM analysis, cotton ovules were placed in tissue fixative consisting of 3% (v/v) glutaraldehyde in 0.1 M sodium phosphate, pH 7.0 and stored at 4°C. The time-points utilized for SEM were -1 to 5 DPA. After fixation, the cotton ovules were dehydrated in a graded ethanol series starting from 20% (v/v) up to 100% (v/v) ethanol. After three changes of 100% ethanol, the ovules were placed in American Optical microporous specimen capsules under 100% ethanol [40] and critical point dried from liquid carbon dioxide by standard methodology in a Ladd Critical Point Dryer model 28,000 (Ladd Research, Williston, VT). The ovules were mounted on standard Cambridge SEM stubs using double-stick Avery photo tabs, #06001. The SEM mounts were coated with 60/40 gold/palladium using a Hummer™ II Sputter Coater (Ladd Research, Williston, VT) to a thickness of 200 nm. The specimens were examined in a XL30 Environmental Scanning Electron Microscope (FEI Company, Hillsboro, OR) at an accelerating voltage from 10-15 kV under high vacuum conditions.

### Cotton fiber total RNA isolation

Cotton fibers were isolated from developing ovules using a glass bead shearing technique to separate fibers from the ovules [41]. Total RNA was isolated from detached fibers using the Sigma Spectrum™ Plant Total RNA Kit (Sigma-Aldrich, St. Louis, MO) with the optional on-column DNase1 digestion according to the manufacturer's protocol. The concentration of each RNA sample was determined using a NanoDrop 2000 spectrophotometer (NanoDrop Technologies Inc., Wilmington, DE). The RNA quality for each sample was determined by RNA integrity number (RIN) using an Agilent Bioanalyzer 2100 and the RNA 6000 Nano Kit Chip (Agilent Technologies Inc., Santa Clara, CA) with 250 ng of total RNA per sample.

### Reverse transcription quantitative real-time PCR

The experimental procedures and data analysis related to RT-qPCR were performed according to the Minimum Information for Publication of Quantitative Real-Time PCR Experiments (MIQE) guidelines [42]. The cDNA synthesis reactions were performed using the iScript™ cDNA Synthesis Kit (Bio-Rad Laboratories, Hercules, CA) according to the manufacturer's instructions with 1 μg of total RNA per reaction used as template. Control cDNA synthesis reactions to check for genomic DNA contamination during RT-qPCR consisted of the same template and components as the experimental reactions without the reverse transcriptase enzyme. The RT-qPCR reactions were performed with iTaq™ SYBR^® ^Green Supermix (Bio-Rad Laboratories) in a Bio-Rad CFX96 real time PCR detection system. Thermal cycler parameters for RT-qPCR were as follows: 95°C 3 minutes, 50 cycles of 95°C 15 seconds, 60°C 30 seconds. A dissociation curve was generated and used to validate that a single amplicon was present for each RT-qPCR reaction. The calculations for amplification efficiencies of the target and reference genes, RNA inhibition assays, and the relative quantifications of the different target gene transcript abundances were performed using the comparative C_q _method as described in the ABI Guide to Performing Relative Quantitation of Gene Expression Using Real-Time Quantitative PCR (Applied Biosystems, Foster City, CA) with the following modification: the average of three reference gene C_q _values was determined by taking the geometric mean which was used to calculate the ΔC_q _values for the individual target genes [43]. The endogenous reference genes used in the RT-qPCR reactions were the 18S rRNA (Genbank accession U42827), ubiquitin-conjugating protein (Genebank AI730710), and alpha-tubulin 4 (*TubA4*, Genbank AF106570) [44]. The reference and target gene primer sequences, and target gene descriptions including *in silico *specificity screens using BLASTx [45] are shown in Additional file 2.

Initial analyses of specific transcripts in fibers of *Li_2 _*mutant and WT plants were performed to determine the most informative time-points to use for microarray analysis. The genes selected were based on previous studies that indicated developmental regulation of these genes during different stages of fiber development, specifically elongation and SCW biosynthesis. The elongation stage-related genes α-expansin1 (*GhExp1*) [46] and Cu/Zn superoxide dismutase (*GhCSD1*) [47] and the SCW-related genes cellulose synthase2 (*GhCesA2*) [48] and a *β-1,3-glucanase-like *gene [49] that was previously shown to be up-regulated in the fiber SCW stage [50] were selected for preliminary RT-qPCR. The nucleotide primer sequences and gene accessions are shown in Additional file 2. The RT-qPCR reactions were performed with cDNA from fibers at initiation, elongation, and SCW thickening stages at the following time-points: DOA, 1, 3, 5, 8, 12, 16, and 20 DPA. Three biological replications were used for each time-point sample.

### Microarray hybridizations and data analysis

The microarray experiments conducted in this study followed the minimum information about a microarray experiment (MIAME) guidelines [51]. The microarray utilized was the commercially available Affymetrix GeneChip^® ^Cotton Genome Array (Affymetrix Inc., Santa Clara, CA), that represents 21,854 transcripts gathered from four species of cotton (*G. arboreum, G. barbadense, G. hirsutum*, and *G. raimondii*) and a variety of tissue types including fibers at the initiation, elongation, and SCW biosynthesis stages of development. For each sample 500 ng of cotton fiber RNA was utilized for labeling using the Affymetrix GeneChip^® ^3' IVT Express Kit and Cotton Genome Array hybridizations were performed according to standardized Affymetrix protocols. The developmental time-points from *Li_2 _*mutant and WT fibers used for microarray hybridizations were 0, 8, and 12 DPA with two biological replicates used for each time-point sample. Procedures for data normalization and assessment of statistically and biologically significant genes were performed as described by Benedito et al. [2008][52]. The Affymetrix microarray dataset was deposited in the ArrayExpress database with the expression number E-MEXP-3306.

To gain insight into the biological processes represented by the differentially expressed genes in the WT and mutant *Li_2 _*fibers, an over-representation analysis (ORA) was performed on the genes that were more abundantly transcribed and statistically significant in fibers of each cotton NIL at each time-point using the program Blast2GO [53]. The Blast2GO program utilizes a Fisher's exact test for ORA and corrects the p-value to control the increased false discovery rate (FDR) associated with multiple hypotheses testing. For this experiment the FDR-corrected p-value cutoff for significance was 0.05.

## Results

### Confirmation of homozygosity in the mutant *Li_2 _*lines

**Figure 1:**
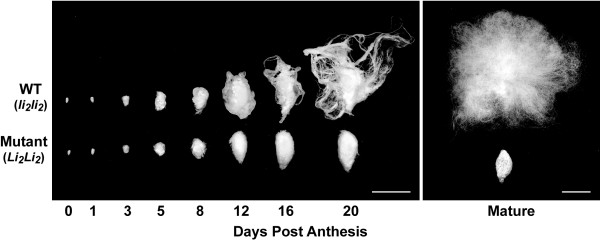
**Comparison of *Li_2 _*mutant and WT fibers and ovules during development**. The differences in development of *Li_2 _*mutant and WT fibers and ovules are shown at selected time-points from DOA to 20 DPA (left panel), and at maturity following boll opening (right panel). The scale bars in both panels are 1 cm.

Segregation analysis of the mutant phenotype was accomplished by visual inspection of the ovules from each one of the 102 plants in the six mutant lines. An example of the *Li_2 _*mutant and WT fiber development stages from the DOA to maturity are shown in Figure 1 with slight differences in fiber length evident as early as 5 DPA. Definitive confirmation of the mutant *Li_2 _*phenotype was possible by 16 DPA and indicated that five of the six lines were homozygous *Li_2_Li_2_*, while one line was heterozygous *Li_2_li_2_*. The heterozygous line was removed from the greenhouse and both the mutant and WT population were reduced to 72 individual plants each to achieve the randomized complete block design of the experiment.

### Morphology of mutant *Li_2 _*fibers during initiation and early elongation

**Figure 2:**
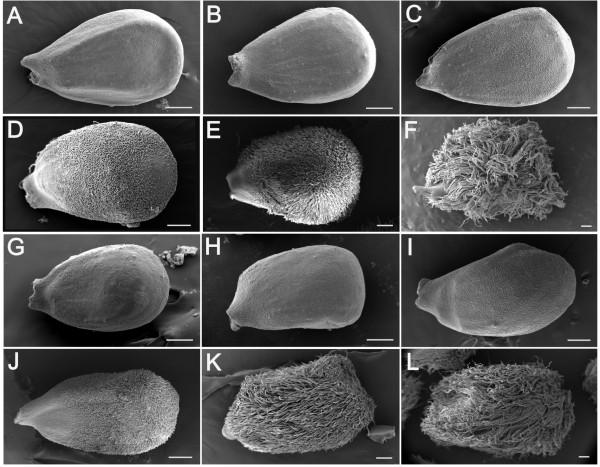
**SEM analysis of developing *Li_2 _*mutant and WT fibers and ovules**. Comparison of (**A - F**) WT; and *Li_2 _*(**G - L**) mutant fibers and ovules prior to and during fiber initiation and early elongation. The developmental time-points shown are: (**A, G**) -3 DPA; (**B, H**) -1 DPA; (**C, I**) DOA; (**D, J**) 1 DPA; (**E, K**) 3 DPA; (**F, L**) 5 DPA. The scale bars in all panels are 200 μm.

SEM analyses revealed no discernable differences in the appearances of ovules and fibers from *Li_2 _*mutant and WT (Figure 2). The WT and mutant fibers appeared to undergo normal initiation as visualized on the DOA with no apparent differences in the distribution or density of fiber initials on the ovule surfaces (Figure 2, C &2I). Likewise, WT and mutant fiber morphology and length appeared the same during the early elongation stage of fiber development up to 5 DPA (Figure 2, D-F; J-L). While no differences between WT and mutant fibers were evident in the SEM (Figure 2), there does appear to be some slight length differences at 5 DPA as shown in Figure 1, so it can be stated that the mutant fiber phenotype becomes evident by approximately 5 DPA. These data are consistent with comparable fiber length measurements obtained from developing fibers of TM-1, *Li_2_*, and F_3 _progeny derived from a cross of TM-1 and *Li_2 _*[28].

### Preliminary RT-qPCR using genes differentially expressed during cotton fiber development

**Figure 3:**
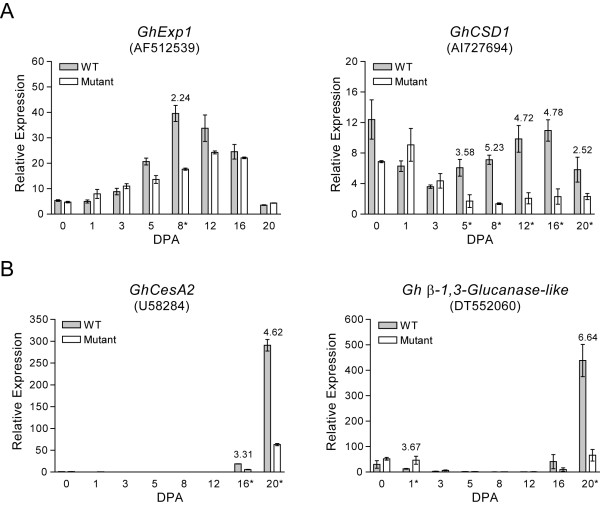
**Preliminary RT-qPCR analysis on developing *Li_2 _*mutant and WT fibers**. Genes more abundantly transcribed during the fiber elongation stage in normal WT tetraploid cotton fibers: (**A**) *G. hirsutum *α-expansin1 (*GhExp1*) and *G. hirsutum *Cu/Zn superoxide dismutase1 (*GhCSD1*). Genes more abundantly transcribed during the fiber SCW stage in normal WT tetraploid cotton fibers: (**B**) *G. hirsutum *cellulose synthase2 (*GhCesA2*) and *G. hirsutum *β-1, 3-glucanase-like gene. Genbank accession numbers are shown in parentheses on the graph titles for each gene. The DPA time-points that revealed a significant (≥ 2-fold; p-value < 0.05) difference in transcript abundance are indicated by an asterisk and the fold-change in transcript abundance is shown on the graphs above each indicated time-point. Error bars indicate the standard deviation from three biological replicates.

The expression of the elongation stage-related gene *GhExp1 *followed a similar pattern of expression in WT and *Li_2 _*mutant fibers, with transcript abundance decreasing at the beginning of the SCW stage from 16 - 20 DPA. However, the transcript abundance of *GhExp1 *was a significantly decreased (2.24-fold) in *Li_2 _*mutant fibers compared to WT fibers during the presumed peak of fiber elongation at 8 DPA. The transcript abundance of the second elongation stage-related gene, *GhCSD1*, followed the previously shown pattern of expression during fiber development with transcript abundance higher during fiber elongation compared to the SCW biosynthesis stage [47]. In fibers of the *Li_2 _*mutant, the expression levels of *GhCSD1 *remained significantly lower compared to WT fibers during elongation stage time-points (5 - 12 DPA) and remained at a seemingly basal level of expression with no change in transcript abundance in mutant fibers over time-points normally associated with elongation and SCW biosynthesis from 5 - 20 DPA (Figure 3A).

The SCW-related genes *GhCesA2 *and *β-1,3-glucanase-like *followed the expected expression profiles in WT and *Li_2 _*mutant fibers with up-regulation occurring concurrent with onset of the transition stage from 12 - 16 DPA and SCW biosynthesis 16 - 20 DPA (Figure 3B). While the pattern of expression for the SCW-related genes was the same in WT and *Li_2 _*mutant fibers, the transcript abundance of both genes was significantly higher in the WT fibers at 16 and 20 DPA for *GhCesA2 *(3.31- and 4.62-fold, respectively) and 20 DPA for the *β-1,3-glucanase-like *gene (6.64-fold) as shown in Figure 3B.

### Microarray gene expression analysis

The initial RT-qPCR analysis indicated significantly different expression levels of the elongation stage-related genes *GhExp1 *and *GhCSD1 *during time-points associated with the elongation stage of fiber development (Figure 3A). Since there were no apparent morphological differences between WT and *Li_2 _*mutant fibers during initiation or early elongation (Figure 2), this microarray study focused primarily on time-points related to fiber elongation, more specifically, 8 and 12 DPA. These time points typically represent peak rates of elongation and the beginning of the transition stage when fibers are still elongating at lower rates. An additional time-point, DOA, was added to the microarray experiment to serve as a reference and also to confirm that there were no significant differences in fiber initiation at the gene expression level. Global gene expression analysis by microarray revealed a significant number of differentially expressed genes in fibers of the mutant and WT NILs at 8 and 12 DPA. At 8 and 12 DPA, there were 1079 and 1106 genes, respectively, more abundantly transcribed (≥ 2-fold; < Bonferroni-corrected p-value threshold 2.07194E-06) in fibers of the WT NIL compared to the mutant fibers. In fibers of the *Li_2 _*mutant NIL 1064 and 1473 genes were significantly and more abundantly transcribed at 8 and 12 DPA, respectively, compared to WT fibers. Far fewer genes were differentially expressed in fibers of the WT and mutant lines in the DOA time-point with 17 genes more abundantly transcribed in WT fibers and 141 genes more abundantly transcribed in mutant fibers.

On the DOA, there were no significantly enriched biological processes represented by the differentially expressed genes in WT or mutant fibers. This result corresponded with the lack of any observable differences in fiber morphology between WT and mutant *Li_2 _*fibers during initiation and early elongation. In 8 DPA fibers of the WT line, over-represented biological processes included, among others, receptor activity, signaling, cytoskeleton, and cell growth, while genes more abundantly transcribed at 8 DPA in mutant *Li_2 _*fibers were enriched in stress and stimulus responses. The genes involved in signaling, cell growth, and cytoskeleton biological processes at 8 DPA in the WT fibers included genes whose products are known to regulate cell elongation and expansion in *Arabidopsis thaliana*. Among these were genes known to encode protein kinases that regulate cell elongation in *A. thaliana *through brassinosteroid (BR) signaling pathways such as orthologues of BRASSINOSTEROID INSENSITIVE1 (BRI1), and HERCULES1 (HERK1) and HERCULES2 (HERK2). Some of the genes that encode proteins with the potential to alter or regulate cell elongation are shown in Table 1 along with the functionally characterized phenotypes resulting from inhibition or over-expression in *A. thaliana*. Genes of interest with higher transcript abundance in *Li_2 _*mutant fibers compared to WT fibers at 8 and 12 DPA, particularly those related to stress and stimulus responses, involved phytohormone signaling pathways for ethylene and cytokinin biosynthesis and regulation of phytohormone homeostasis. The potential implications of genes and biological processes over-represented in WT and *Li_2 _*mutant fibers during elongation are discussed later and summarized in Table 1. The complete results of the ORA analysis are presented in Additional file 3 which includes the individual microarray probeset IDs for the over-represented biological processes for each cotton NIL and time-point.

**Table 1:** Heterologous functions of genes differentially expressed in *Li_2_*WT and mutant fibers during elongation

Gene more abundantly expressed in *Li_2 _*WT fibers compared to mutant fibers				
**Microarray Probesets ID**	**Sequence Desciption and Locus Tag**	**Genbank**	**Mutant Phenotype**	**Reference**

Ghi.3835.2.S1_s_at	*A. thaliana *TRH1 (TINY ROOT HAIR 1). Functions as a potassium transporter and is required for the establishment of root tip growth. AT4G23640.	NP_194095	T-DNA insertion. Initiation sites for root hair growth are formed at trichoblasts but tip growth does not occur.	[68]
Ghi.8115.1.S1_s_at	*G. hirsutum *transcription factor GhMYB109. Fiber-specific R2R3 MYB transcription factor highly expressed during elongation.	AY366352	Antisense suppression. Transgenic plants have impaired fiber initials that are smaller and shrunken compared to WT. Fiber elongation is inhibited in the transgenic plants. Mature fibers of transgenic plants have a short-fiber phenotype compared to WT with an ~33% reduction in fiber length.	[75,76]
Ghi.8448.1.S1_x_at	*G. hirsutum *Beta tubulin1 (BTub1): family of small, globular proteins that form microtubules. Active component of the cytoskeleton.	AF521240	Over-expression phenotype. Inducible expression in fission yeast resulted in longitudinal cell growth compared to un-induced cell and empty expression vector controls.	[77]
GhiAffx.24518.1.S1_s_at	A. thaliana BRASSINOSTEROID INSENSITIVE 1 (BRI1): Encodes a plasma membrane localized leucine-rich repeat receptor kinase involved in brassinosteroid signal transduction. AT4G39400.	NP_195650	EMS mutagenesis; T-DNA insertion. Short plant stature and small, dark green leaves. Shortened length of stem and pedicel epidermal cells. Reduced fertility. Reduced apical dominance. De-etiolation of dark-grown seedlings. Allelic bri1 mutants can be divided into severe, intermediate, and weak phenotypes.	[72,86,87]
GhiAffx.36984.1.S1_s_at	*A. thaliana *HERCULES1 (HERK1), a receptor kinase regulated by Brassinosteroids and required for cell elongation during vegetative growth. Functions redundantly with THESEUS1 (THE1). AT3G46290.	Q9LX66	T-DNA insertion. Similar to WT in the herk1 mutant. In the herk1 the1 double mutant, petioles are reduced in length by half and petiole cells are shortened. Over-expression of HERK1 increased petiole length by 15-20%.	[66]
GhiAffx.47644.1.S1_at	A. thaliana HERCULES2 (HERK2), a receptor kinase regulated by Brassinosteroids and required for cell elongation during vegetative growth. Functions redundantly with HERCULES1 (HERK1) and THESEUS1 (THE1). AT1G30570.	NP_174345	T-DNA insertion. Similar to WT in the herk2 mutant. In the herk1 herk2 the1 triple mutant, petioles are further reduced in length compared to the herk1 the1 double mutant.	[67]
GhiAffx.62092.1.S1_at	*A. thaliana *XIK: Member of the type XI myosin protein family. Involved in root hair growth, trichome development, and organelle trafficking. AT5G20490.	NP_001154724	T-DNA insertion. Normal root growth but root hairs are reduced in length by 50%. Leaf and stem trichomes have size and shape irregularities.	[69]
GhiAffx.6933.1.A1_at	*A. thaliana *CROOKED (CRK): Belongs to the DIS (distorted) gene family. Encodes an actin polymerization factor. Part of the Arpc2/3 protein complex as ARPC5. Involved in cell expansion of trichomes. AT4G01710.	NP_567216	EMS mutagenesis. Shortened leaf trichomes and trichome branches. Reduced polarized expansion of hypocotyls. Cells of expanding hypocotyls are shorter.	[70]
Gra.2039.1.S1_s_at	*A. thaliana *COBRA (COB): Glycosylphosphatidylinositol (GPI)-anchored protein localized primarily in the plasma membrane of the longitudinal sides of root cells. Necessary for oriented cell expansion in Arabidopsis. AT5G60920.	NP_568930	EMS mutagenesis; x-ray mutagenesis; T-DNA insertion. Increased root diameter and short roots due to increased width and reduced length of cells. Reduced root cell division rate and change in the orientation of root expansion.	[71,88]
				
Gene more abundantly expressed in *Li_2 _*mutant fibers compared to WT fibers				

Microarray Probesets ID	Sequence Description and Locus Tag	Genbank	Mutant Phenotype	Reference

Ghi.7529.1.A1_s_at; Ghi7529.1.S1_s_at; Gra.511.1.A1_s_at	*A. thaliana *AHP1 (HISTIDINE-CONTAINING PHOSPHOTRANSMITTER 1): AHPs function as redundant positive regulators of cytokinin signaling. AT3G21510.	NP_188788	T-DNA insertion. Loss of root elongation sensitivity in response to exogenous application of cytokinin.	[80]
GhiAffx.12783.1.S1_s_at	*A. thaliana *homeobox protein HAT22: member of the HD-Zip II family of transcription factors. AT4G37790.	NP_195493	Mutant phenotype not described. Up-regulated in response to exogenous cytokinin application in A. thaliana WT and cytokinin-deficient mutant plants	[81]
Ghi.3235.1.A1_at	*A. thaliana *UDP-glucosyl transferase 73C2 (UGT73C2): Involved in cytokinin N-glucosylation. Along with UGT73C1, glucosylates all cytokinins at the N7 and N9 positions. AT2G36760.	DT463078	Over-expression phenotype. Plants constitutively over-expressing UGT73C1 were generated, but no phenotype was reported.	[82]
Ghi.9236.1.S1_at	*A. thaliana *UDP-glycosyltransferase 73C5 (UGT73C5)/DON-GLUCOSYLTRANSFERASE 1 (DOGT1): Glucosylates brassinolide and castasterone in the 23-O position. Presumably involved in the homeostasis of those steroid hormones and regulation of BR activity. Also shown to glucosylate the OH group on the N6-side chain of the cytokinins trans-zeatin and dihydrozeatin. AT2G36800.	DT463077	Over-expression phenotype. Plants over-expressing UGT73C5/DOGT1 displayed typical BR-deficient dwarf phenotypes such as reduced hypocotyl elongation, and contained reduced levels of BRs.	[82,83]
Ghi.4551.1.S1_at	*A. thaliana *SUPPRESSOR OF BIR1 (SOBIR1): Encodes a leucine rich repeat transmembrane protein that is a receptor-like kinase (RLK). SOBIR1 is suggested to be a critical positive regulator of cell death. AT2G31880.	Q9SKB2	Over-expression phenotype. Constitutive over-expression of sobir1 results in activation of cell death.	[89]

### Corroboration of the microarray data

A total of twelve genes were selected for corroboration of the microarray data, in addition to the cotton Cu/Zn superoxide dismutase (*GhCSD1*) gene that was utilized for preliminary RT-qPCR data to select fiber developmental time-points for microarray analysis (Figure 3). Of the four genes selected for the preliminary RT-qPCR, only the *GhCSD1 *gene is accurately represented in the Affymetrix microarray data with the probeset ID Ghi.2036.1.S1_s_at (Table 2, Additional file 2). The *GhCesA2 *gene shares 100% homology with the probesets nucleotide sequence, but is below the limit of detection during the initiation and elongation stages selected for microarray analysis. The *GhExp1 *and *β-1,3-glucanase-like *genes used to design primers for RT-qPCR (Additional file 2) do not share enough homology with the Affymetrix nucleotide sequences in the regions corresponding to the probsets to allow for accurate comparison of transcript abundance between microarray and RT-qPCR.

**Table 2:** Expression ratios and statistical significances from the microarray and RT-qPCR data

Microarray		DOA (WT/Mutant)		8 DPA (WT/Mutant)		12 DPA (WT/Mutant)	
		
Probesets ID	Blastx Sequence Description	Microarray	RT-qPCR	Microarray	RT-qPCR	Microarray	RT-qPCR
Ghi.2036.1.S1_s_at	Cu/Zn superoxide dismutase (GhCSD1)	1.48*	1.80*	2.52*	5.22*	1.78*	4.72*
Ghi.1711.1.S1_s_at	Putative SANT/MYB Transcription Factor	0.80*	0.51*	0.19*	0.65	0.16*	0.34*
Ghi.3235.1.A1_at	Putative UDP-glycosyltransferase (UGT73C2)	0.72*	0.53	0.03*	0.0077*	0.23*	0.033*
Ghi.4377.1.A1_at	Glycuronosyltransferase-like protein	0.78*	0.67	3.83*	7.85*	1.20*	2.67*
Ghi.6551.1.S1_at	BZIP domain class transcription factor	0.72*	0.57*	1.90*	2.59*	2.94*	1.84*
Ghi.7279.1.S1_at	Putative ABC transporter	1.43*	1.22*	3.82*	6.86*	6.03*	5.44*
Ghi.7450.1.S1_s_at	ECERIFERUM 3 (CER3)	0.79*	0.81	2.31*	2.35*	3.17*	1.64*
Ghi.7724.1.S1_at	Cellulose-synthase-like C5 (AtCSLC5)	0.98*	0.67*	2.44*	5.96*	4.37*	3.97*
Ghi.8115.1.S1_s_at	Transcription factor GhMYB109	0.97*	0.71*	5.02*	3.45*	7.80*	4.67*
Ghi.8665.1.S1_s_at	Sucrose synthase	1.17	1.05	0.72	0.53	1.11	0.64
Ghi.9209.1.S1_at	Putative R2R3-Myb transcription factor	0.94*	1.30	0.27	0.60	0.53	1.00
Ghi.9236.1.S1_at	Putative UDP-glycosyltransferase (UGT73C5/DOGT1)	0.92*	0.25*	0.08*	0.0035*	0.55	0.0083*
Gra.2056.1.A1_s_at	Beta-galactosidase	0.41*	0.13	39.90*	101.53*	41.57*	38.62*

* Microarray data: significant at the Bonferroni-corrected 0.05 probability level; RT-qPCR data: significant at the 0.05 probability

level as determined by two-tailed paired t-test for means. Microarray and RT-qPCR data significant at ≥ 2-fold difference in transcript abundance are shown in boldface and underlined.

The genes selected for microarray corroboration by RT-qPCR were chosen primarily from differentially expressed genes (≥ 2-fold; < Bonferroni-corrected p-value threshold 2.07194E-06) in WT and mutant fibers at 8 and 12 DPA. Some selected genes were more abundantly expressed at 8 and/or 12 DPA in WT fibers, while others were more abundantly expressed in mutant fibers at the same time-points. These genes encode regulatory elements such as transcription factors including the *G. hirsutum *fiber initiation-related MYB transcription factor GhMYB109; gene products involved in carbohydrate metabolism such as beta-galactosidase, glycosyltransferases; and a cellulose-synthase-like cell wall-related protein. Genes that were not differentially expressed during elongation time-points were also selected to confirm the validity of the microarray data. These included genes encoding a R2R3-MYB transcription factor (probeset ID Ghi.9209.1.S1_at) and sucrose synthase (probeset ID Ghi.8665.1.S1_s_at). The microarray data were successfully corroborated for most time-points with the exception of the SANT/MYB transcription factor gene (probeset ID Ghi.1711.1.S1_s_at) at 8 DPA. The complete results of the microarray data and the corresponding RT-qPCR data for all twelve genes and the *GhCSD1 *gene are summarized in Table 2 and Additional file 4.

### Fine mapping the *Li_2 _*locus region with codominant SSR markers

**Figure 4:**
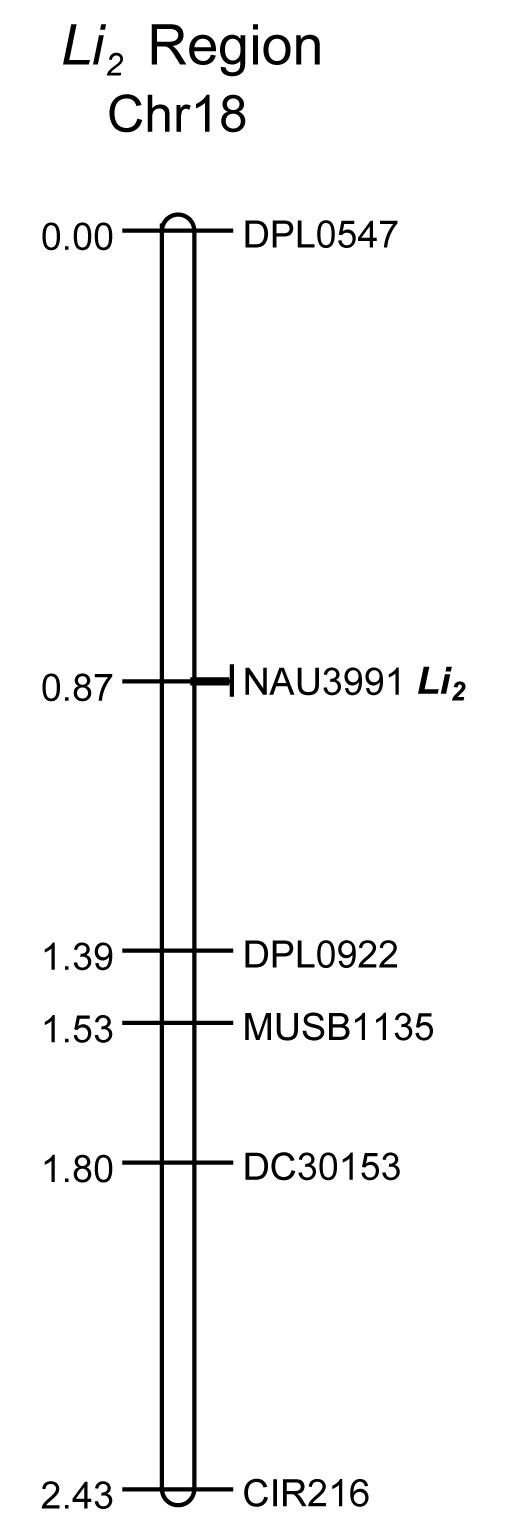
**Genetic linkage map of the *Li_2 _*locus on chromosome 18 of *G. hirsutum***. The linkage map indicates SSR markers flanking a 2.43 cM region of the *Li_2 _*locus. The EST-SSR marker NAU3991 with complete linkage to the *Li_2 _*locus is indicated on the map by *Li_2_*.

Of the 86 SSR markers screened, 6 (6.97%) were polymorphic between two DNA bulks from *Li_2 _*mutant and WT plants. Except for the marker TMB2295 that had 5 recombinations with the *Li_2 _*trait when the 20 individuals comprising the bulks were tested, the markers CIR216, DC30513, DPL0547, DPL0922, and MUSB1135 co-segregated with the *Li_2 _*trait. These 5 markers were further evaluated in the whole F_2 _population. A map was constructed around the *Li_2 _*region (Figure 4). The *Li_2 _*locus was flanked by markers DPL0547 and DPL0922 at genetic distances of 0.87 cM and 0.52 cM, respectively.

### Identification of an EST-SSR marker with complete linkage to the *Li_2 _*locus

In an effort of using gene expression data to identify candidate molecular markers for association mapping of the *Li_2 _*gene, we conducted a BLAST search [54] of the Cotton Marker Database (CMD) (http://www.cottonmarker.org) using consensus sequences and singletons representing differentially expressed genes from the microarray data. The search criteria were set at a stringent level (e-value > E-50) in order to select sequences with a relatively high sequence alignment length and homology to the microarray query sequences. Query sequences included genes more abundantly expressed (≥ 2-fold) in fibers of both mutant and WT cottons at 8 and 12 DPA. The numbers of query sequences from WT fibers were 1031 and the numbers of query sequences from mutant fibers were 982. A total of 692 SSR markers were identified based on sequence homology between the microarray query nucleotide sequences and the SSR source nucleotide sequences. Of these 692 SSR markers, 179 were previously mapped by different research groups [31,33-35,55], and 14 of them were mapped on chromosome 18 or 13. Except markers DPL0249 and TMB1767 that were developed from genomic DNA libraries, the other 12 were EST-SSRs (http://www.cottonmarker.org). We analyzed these 14 SSR markers in the F_2 _population, and only one (NAU3991) segregated among progeny plants. The marker NAU3991 was developed from *G. raimondii *EST [31] and displayed complete linkage to the *Li_2 _*genetic locus in our intraspecific F_2 _population composed of 136 individuals (Figure 4). The Blastx hits for the full-length cDNA with highest similarity in the NCBI non-redundant database included a putative transcription factor isolated from *Ricinus communis*; plectin-related proteins from *A. thaliana *and *Vitis vinifera*; and unknown proteins from *Populus trichocarpa *and *Glycine max*. The full-length predicted protein sequences are highly conserved among cotton and the other plant species for all of the above mentioned protein sequences. The NCBI conserved domain database (CDD) [56] indicated a putative DNA binding domain present in the predicted protein sequence.

**Figure 5:**
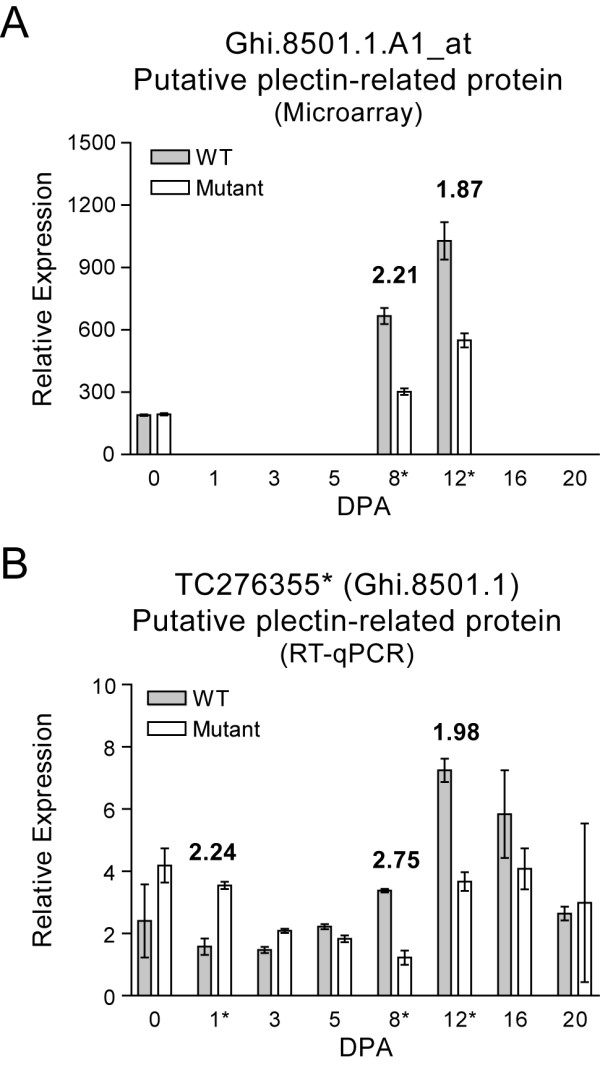
**Microarray and RT-qPCR gene expression profiles of the *G. hirsutum *gene harboring the NAU3991 EST-SSR marker**. The cotton gene with the NAU3991 EST-SSR marker is designated "putative plectin-related protein" based on sequence similarity to a putative *A. thaliana *orthologue. (**A**) The transcript abundance of the gene in *Li_2 _*mutant and WT fibers as determined by microarray. Relative expression represents signal intensity of probesets. Error bars indicate standard deviation from 2 biological replicates. The DPA time-points that revealed a significant (≥ 2-fold; < Bonferroni-corrected p-value threshold 2.07194E-06) are indicated by an asterisk and the fold-change in transcript abundance is shown on the graph above each indicated time-point. (**B**) The transcript abundance of the gene in *Li_2 _*mutant and WT fibers as determined by RT-qPCR. Error bars indicate standard deviation from 3 biological replicates. The gene is represented by the indicated TC in the Cotton Gene Index 11.0 database (http://compbio.dfci.harvard.edu/tgi/). The DPA time-points that revealed a significant (≥ 2-fold; p-value < 0.05) difference in transcript abundance are indicated by an asterisk and the fold-change in transcript abundance is shown on the graphs above each indicated time-point.

The microarray probeset that led to the discovery of the linkage between NAU3991 and *Li_2 _*locus, Ghi.8501.1.A1_at was represented as TC276355 in the Dana-Farber Cancer Institute Cotton Gene Index (DFCI) 11.0 database http://compbio.dfci.harvard.edu/tgi/. The RT-qPCR primers used to corroborate the microarray data for probeset Ghi.8501.1.A1_at was designed from TC276355 (Additional file 2). The expression profile of TC276355 matched the microarray data for probesets Ghi.8501.1.A1_at and indicated that the gene was up-regulated in WT fibers during the fiber elongation and transition stages (8 - 16 DPA) with significantly higher transcript abundance in WT fibers compared to mutant fibers at 8 and 12 DPA (Figure 5).

## Discussion

Naturally occurring cotton fiber mutations present an excellent opportunity to study the molecular events controlling fiber development. When a monogenic fiber mutation in the homozygous state is coupled with its homozygous WT counterpart, especially as NILs, an ideal model system is created for a comparative study of fiber development. Here we have presented such a model system in the *Li_2 _*short-fiber mutation as an advanced (BC_5_) NIL in the *G. hirsutum *DP5690 background. In addition to the *Li_2 _*short-fiber mutant phenotype being caused by a single dominant allele, no pleiotropic effects are observed in the development, morphology, or fecundity of mutant cotton plants as either homozygotes or heterozygotes. Also, no pleiotropic effects of the *Li_2 _*mutation are observed in mutant fiber development before the elongation stage. In this regard, the *Li_2 _*mutant is useful to study molecular events specific to and controlling fiber elongation.

It was previously demonstrated that the fibers of another dominant, monogenic short-fiber mutation similar to *Li_2_*, designated *Li_1_*, have a thicker SCW than WT fibers and *Li_2 _*mutant fibers. This was demonstrated by measuring the ratio of fiber weight-to-length to infer the degree of SCW thickening in fibers of *Li_1_*, *Li_2_*, TM-1, and normal homozygous recessive *Li_2 _*segregates (F_3_) from a cross between *Li_2 _*and TM-1 [28]. The thickened SCW of *Li_1 _*fibers was further confirmed in another study that measured the incorporation of [^14^C] glucose in the SCW and indicated a five-fold increase in crystalline cellulose synthesis per millimeter of fiber in the *Li_1 _*mutant compared to WT fibers [57]. This suggests that the pleiotropic effect of the short lint fiber *Li_1 _*mutation also affects multiple stages of fiber development, while the *Li_2 _*mutation remains specific to fiber length. It was also speculated that the thickened SCW walls of *Li_1 _*fibers may be due to increased SCW cellulose synthase activity or an increased density of cellulose synthase subunits per unit length of fiber causing an increase in cellulose deposition in the SCW [57]. The later speculation would likely result in an increase in the transcript abundances of SCW *CesA *genes such as *GhCesA2 *in *Li_1 _*mutant fibers compared to WT fibers during the SCW stage. A recent comparative gene expression study on the SCW stage of *Li_1 _*mutant and WT TM-1 fibers would have provided insight into this theory, however the SCW *CesA *genes were not discussed and the microarray dataset was not made publically available [12]. The authors did report 8.8-fold higher transcript levels of a sucrose synthase (*SuSy*) gene (Genbank accession U73588) in *Li_1 _*mutant fibers compared to WT TM-1 fibers that could support the observed increased cellulose deposition in the SCW of *Li_1 _*mutant fibers [12,57]. Our RT-qPCR data for the SCW-related genes *GhCesA2 *and *β-1,3-glucanase-like *indicated significantly higher levels of expression at the beginning of SCW synthesis in WT fiber compared to *Li_2 _*mutant fibers (Figure 3B). This would be the converse of the *Li_1 _*fibers if the density of *CesA *subunits per unit length of fiber were higher in *Li_1 _*mutant fibers compared to WT fibers and supports the fiber elongation-specific, non-pleiotropic nature of the *Li_2 _*mutation. The *SuSy *gene that was selected as a non-differentially expressed gene for microarray corroboration also supports this hypothesis (Table 2; Additional file 4). The RT-qPCR on all developmental time-points in our study using primers specific for the *G. hirsutum SuSy *gene (Genbank accession U73588) matched the *GhCesA2 *expression at 20 DPA (Figure 3B) with statistically significant 2.54-fold higher *SuSy *transcript abundance in WT fibers compared to mutant fibers (Additional file 4).

The significantly higher expression levels of the *GhCSD1 *gene in WT fibers compared to mutant fibers coincided well with a previously postulated model that suggested a role for the modulation of ROS in fiber cell elongation. The model suggested that short fiber cotton species such as the diploid *G. longicalyx *are unable to regulate ROS accumulation during elongation, leading to stress conditions, an increase in the expression of stress-related genes, and a decrease in the rate of fiber cell elongation [58]. Comparison of the fiber gene expression profiles of *G. longicalyx *and diploid *G. arboreum *during elongation (5 DPA) revealed over-representation of biological processes involved in response to stress, presumably due to over-accumulation of ROS, in the shorter fibers of *G. longicalyx*. Results indicating the same differences in ROS homeostasis were obtained by the same research laboratory in two more microarray gene expression studies that compared short and long fiber cotton species during elongation [59,60]. A similar over-representation of stress response-related genes (GO:0006950) was observed in *Li_2 _*mutant fibers compared to WT fibers at 8 DPA in our study, including genes responsive to oxidative stress and involved in ROS homeostasis (Additional file 3). A total of 105 genes were included in the over-representation of stress responses in *Li_2 _*mutant fibers at 8 DPA and included ROS-related genes such as an NADPH-dependent aldo-keto reductase (AKR) with high similarity to the *A. thaliana *AKR4C9 protein involved in the reduction of reactive α,β-unsaturated carbonyls produced through lipid peroxidation [61]; an alternative oxidase highly similar to the stress-responsive *A. thaliana *ALTERNATIVE OXIDASE 1A (AOX1A); a *G. hirsutum *ascorbate peroxidase (GhAPX1) involved in H_2_O_2 _homeostasis [62]; a *G. hirsutum *class III peroxidase (GhPOX1) suggested to play a role in ROS production [63]; a transcription factor highly similar to the *A. thaliana *MULTIPROTEIN BRIDGING FACTOR 1C (AtMBF1C) that is over-expressed in response to multiple stimuli including elevated H_2_O_2 _levels [64]; and several putative APETELA2 (AP2)-domain transcription factors that are also up-regulated in transgenic *A. thaliana *over-expressing *AtMBF1C *[65]. Given that some of these gene products are involved in the production of ROS required for normal fiber cell elongation, while others are involved in the reduction oxidative stress and repair of oxidative damage, it is possible that the mechanisms of ROS modulation and homeostasis are compromised in *Li_2 _*mutant fibers.

Numerous genes involved in biological processes such as cell expansion and elongation that are up-regulated in the WT fibers compared to mutant fibers during the time-points coinciding with peak rates of fiber elongation. Genes that were more abundantly transcribed (≥ 2-fold) and statistically significant in either *Li_2 _*mutant or WT fibers during elongation were closely examined to discern the possible effects of their up- or down-regulation based on functional analysis in cotton and heterologous systems. Some examples of these genes with high similarity to their *A. thaliana *orthologues are briefly discussed. Detailed descriptions of these genes are also shown in Table 1. The *A. thaliana *knockout mutants for many of these genes developed by either T-DNA insertion, ethyl methanesulfonate (EMS) mutagenesis, or x-ray mutagenesis suggest roles in cell expansion and elongation. Some examples of the genes more abundantly transcribed in WT fibers during elongation included putative *A. thaliana *orthologues that when silenced by mutagenesis caused shortened petiole cells in *herk1 theseus1 *(*the1*) double mutants [66] and *herk1 herk2 the1 *triple mutants [67]; inhibition of root hair tip growth in mutants for the potassium transporter TINY ROOT HAIR 1 (TRH1) [68]; a 50% reduction in root hair length in mutants for a type XI myosin protein, XIK [69]; shortened leaf trichomes, reduced polarized expansion of hypocotyls, and shorter hypocotyl cells in mutants for the actin polymerization factor CROOKED (ARPC5) [70]; reduced length of root cells in mutants for the glycosylphosphatidylinositol (GPI)-anchored protein COBRA [71]; and reduced cell sizes resulting in dwarfism in the mutant *brassinosteroid insensitive 1 *(*bri1*) [72]. The functionality of a cotton orthologue of BRI1 was previously demonstrated by complementation to rescue the WT phenotype of an *A. thaliana bri1 *mutant. Furthermore, chemical inhibition of a BR mediated response was demonstrated to inhibit fiber initiation and elongation in *in vitro *cotton ovule cultures and *in planta *[73,74]. Two more *G. hirsutum *genes encoded the transcription factor GhMYB109 and the microtubule protein Beta tubulin1 (GhTub1) more abundantly in WT fibers compared to mutant fibers. Antisense suppression of *GhMYB109 *in transgenic cotton caused smaller and shrunken fibers initials and inhibition of fiber elongation resulting in a short-fiber phenotype [75,76]. The *GhTub1 *gene was transcribed 46-fold and 7-fold higher in WT fibers at 8 and 12 DPA, respectively, and resulted in longitudinal cell growth when over-expressed in fission yeast cells under the control of an inducible promoter [77].

It is well-documented that increased levels of natural and/or synthetic cytokinins inhibit cotton fiber cell elongation in the *in vitro *cotton ovule culture system [78,79]. Genes that were more abundantly transcribed in *Li_2 _*mutant fibers compared to WT fibers encode proteins that are involved in cytokinin signaling and included putative orthologues of the *A. thaliana *HISTIDINE-CONTAINING PHOSPHOTRANSMITTER 1 (AtAHP1) that functions as a positive regulator of cytokinin signaling [80]; the *A. thaliana *homeobox domain transcription factor HAT22 that is up-regulated in response to exogenous application of cytokinins [81]; and orthologues of the *A. thaliana *UDP-glycosyltransferases UGT73C2 and UGT73C5/DON-GLUCOSYLTRANSFERASE 1 (AtDOGT1), glycosylate cytokinins which are implicated in cytokinin homeostasis [82]. Over-expression of UGT73C5/*DOGT1 *in transgenic *A. thaliana *results in a dwarf phenotype with reduced elongation of hypocotyls similar to BR-deficient mutants [83]. Increased cytokinin levels as determined indirectly by enzyme-linked immunosorbent assay measurements were previously reported in the ovules and fibers of four cotton fiber mutants compared to WT [84]. This study included Ligon lintless cotton, but did not specify *Li_1 _*or *Li_2_*. While immunoassays that measure plant hormone are generally semi-quantitative, the results do coincide with our gene expression results and suggest a possible role for cytokinin signaling and homeostasis in the *Li_2 _*mutant phenotype (Table 1). Any one of these genes or gene families could plausibly explain the *Li_2 _*short-fiber phenotype based on extensive functional characterization in cotton and heterologous systems such as *A. thaliana*. However, the fact that *Li_2 _*is a monogenic dominant trait suggests that the differential expression of these genes in *Li_2 _*mutant and WT fibers are downstream effects of a defect in a regulatory element controlling fiber development, specifically elongation in the case of the *Li_2 _*mutation.

Little information is available regarding the cotton gene harboring the NAU3991 EST-SSR marker with complete linkage to the *Li_2 _*locus. The function of this gene in plants is speculative and could be structural or regulatory in nature based on the putative DNA binding motif and homology to the mammalian plectin protein that acts as a microfilament crosslinker in mammalian systems [85]. Presently, no functional information is available for this gene in any plant species and no phenotypes listed for mutants of *A. thaliana *or other plant species.

One of the major limitations of a gene expression analysis study is the lack of empirical evidence for functionality of any of the identified candidate genes that may cause an aberrant phenotype or enhance a trait of interest. This leads to a great deal of speculation as we have done here and in other cotton gene expression studies. This is especially true for a quantitative trait such as cotton fiber quality. The main goal of this gene expression study focused on practical concerns such as conversion of gene expression data into quantifiable, portable, and useable systems such as molecular markers. In regards to the regulation of a complex and multi-tiered event such as cotton fiber development by a single dominant gene, it should be expected that many downstream events from the developmental point of global regulation will be dramatically altered resulting in a high false discovery rate of candidate genes that truly are differentially expressed between, for example, mutant and WT fibers, but may be acting synergistically to cause the mutant phenotype. Since *Li_2 _*is a monogenic dominant trait, it is possible that not one of these genes alone is the actual mutant allele causing the phenotype. The discovery of an EST-SSR marker based on gene expression data and with complete linkage to the *Li_2 _*locus presents a novel approach for data mining a genetic marker database. Presently, we are cloning the full-length cDNAs and genomic DNAs for the NAU3991 marker gene from the *Li_2 _*mutant and WT plants and have begun screening a *G. hirsutum *bacterial artificial chromosome (BAC) library. Identification of BAC clones containing the NAU3991 marker gene(s) will allow analysis of the genomic regions flanking the *Li_2 _*locus and facilitate functional analysis of the *Li_2 _*gene in transgenic cotton.

## Conclusion

The cotton short fiber mutation Ligon lintless-2 (*Li_2_*) appears specific to the elongation stage of fiber development with no apparent morphological differences between WT and mutant fibers until approximately 5 DPA. Comparative microarray gene expression profiling identified genes differentially expressed between WT and mutant fibers and suggested a role for ROS and cytokinin regulation that could result in the short fiber phenotype observed in the *Li_2 _*mutant fibers. The microarray gene expression data was successfully converted into an EST-derived SSR marker, NAU3991, which displayed complete linkage to the *Li_2 _*locus on chromosome 18. The complete linkage suggested that the gene harboring the NAU3991, or another unknown gene closely linked to the EST-SSR marker, may be the *Li_2 _*gene. The function of the gene harboring the NAU3991 marker is unknown in plant species, but shares homology with a gene encoding a plectin protein that acts as a microfilament crosslinker in mammalian systems.

## List of Abbreviations

BC: backcross; DOA: day of anthesis; DPA: days post-anthesis; EST: expressed sequence tag; NIL: near-isogenic line; PCW: primary cell wall; RFLP: restriction fragment length polymorphism; ROS: reactive oxygen species; RT-qPCR: reverse transcription quantitative polymerase chain reaction; SCW: secondary cell wall; SEM: scanning electron microscopy; SNP: single nucleotide polymorphism; SSD: single seed decent; SSR: simple sequence repeat; TC: tentative consensus; UTR: untranslated region; WT: wild-type.

## Authors' contributions

DJH had the main responsibility for the study including the greenhouse experiment; tagging and sample harvest; RNA isolations; RT-qPCR and statistical analysis of the RT-qPCR data; gene selection from the microarray data for SSR data mining; and nucleotide sequence analysis. RBT developed the *Li_2 _*mutant and WT NILs and the F_2 _mapping population used as plant material. MN performed RNA isolations and assessment of RNA quality; gene selection for corroboration of the microarray results; RT-qPCR and statistical analysis of the RT-qPCR data; and nucleotide sequence analysis. HJK prepared samples for SEM analysis. YT performed statistical analysis on the microarray data. KMY designed the greenhouse experiment for proper experimental design and statistical analysis of the data. PL assisted with the greenhouse experiment; molecular marker analysis; and conducted cDNA cloning for sequence analysis. DDF conceived the experiment, coordinated and supervised the research, conducted molecular marker analysis, association mapping, and linkage map construction. DJH and DDF wrote the manuscript. All authors read and approved the final manuscript.

## Supplementary Material

Additional file 1 **Pedigree of the *Li_2 _*mutant and WT NILs**. The NILs utilized in this study were in the *G. hirsutum *cv. DP5690 genetic background and in the BC_5 _generation. Click here for file

Additional file 2 **Primer pair sequences designed for marker analysis and RT-qPCR**. The forward and reverse nucleotide primer sequences are shown along with the microarray probesets IDs, BLASTx sequence descriptions, and relevant accession numbers. Click here for file

Additional file 3 **Over-representation analysis (ORA) of genes differentially expressed in *Li_2 _*WT and mutant fibers ≥ 2-fold**. The original p-values for the ORA are shown along with the FDR-corrected and FWER-corrected p-values. The statistical significance cutoff for the ORA was an FDR-corrected p-value of 0.05. The TestSeqs are the microarray probesets IDs over-represented for the indicated biological process. Click here for file

Additional file 4 **Corroboration of the microarray gene expression data by RT-qPCR on *Li_2 _*mutant and WT fibers**. Gene expression profiles of twelve genes selected to verify the microarray gene expression data. Affymetrix probeset IDs and predicted gene products are shown on the graph titles for each gene. The DPA time-points that revealed a significant (≥ 2-fold; p-value < 0.05) difference in transcript abundance are indicated by an asterisk and the fold-change in transcript abundance is shown on the graphs above each indicated time-point. Error bars indicate standard deviation from 3 biological replicates. Click here for file
