# Supplementary figures and images for: A combined functional and structural genomics approach identified an EST-SSR marker with complete linkage to the Ligon lintless-2 genetic locus in cotton (Gossypium hirsutum L.)
Source: BMC Genomics. 2011 Sep 9;12:445. doi: 10.1186/1471-2164-12-445 (PMC3175229; doi:10.1186/1471-2164-12-445)

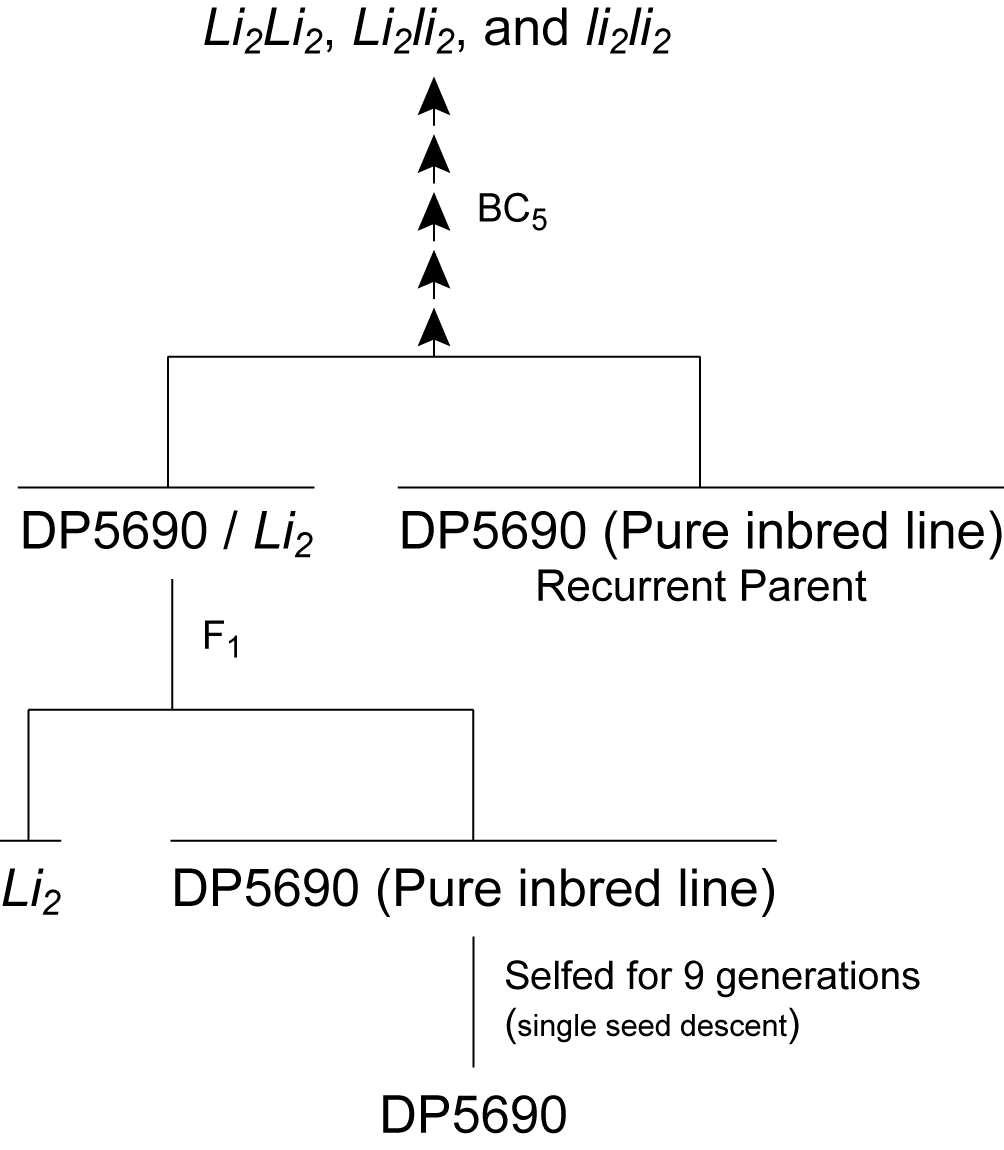

Supplement: Additional file 1 — Pedigree of the Li2 mutant and WT NILs. The NILs utilized in this study were in the G. hirsutum cv. DP5690 genetic background and in the BC5 generation. [file 1471-2164-12-445-S1.TIFF]

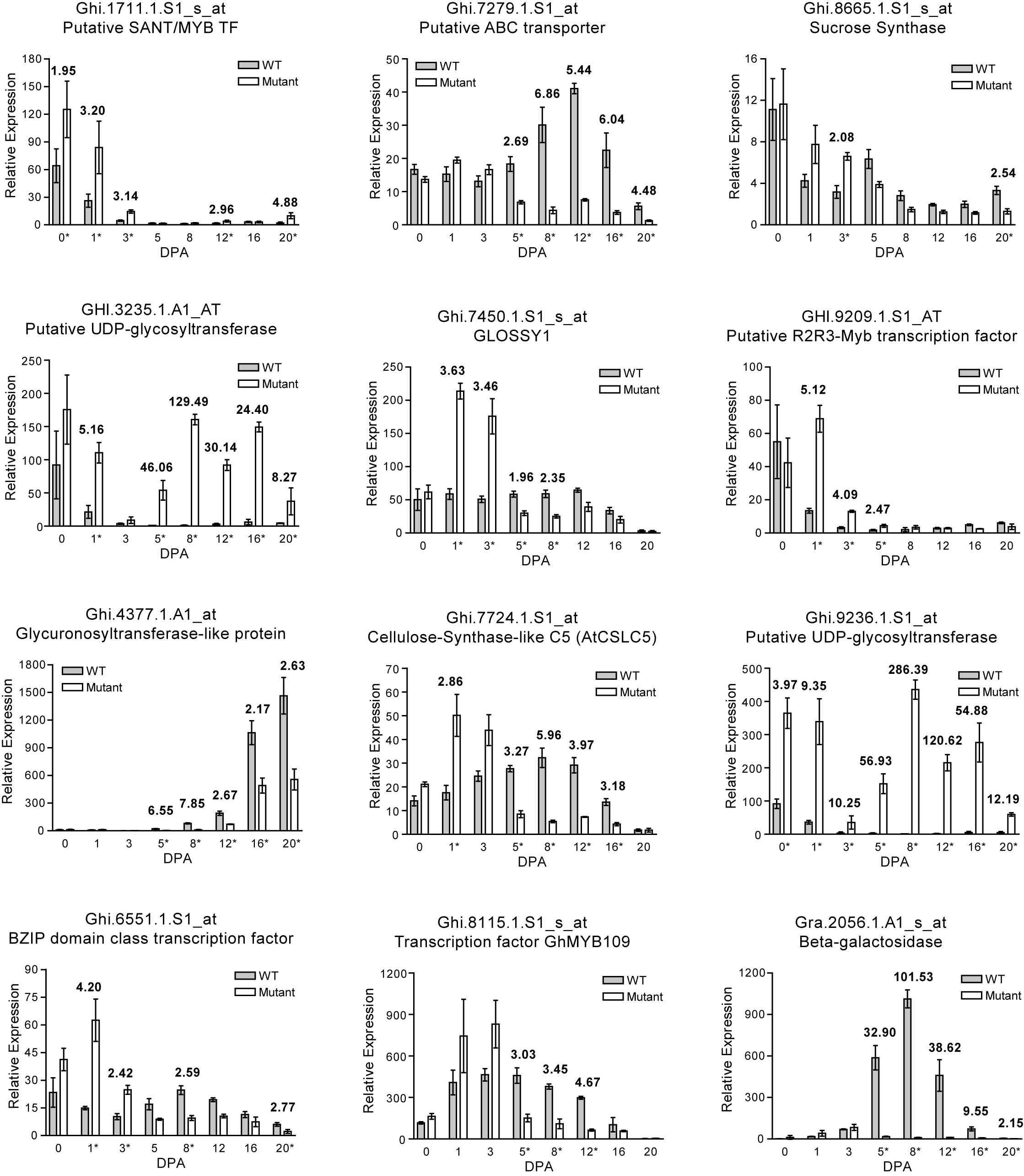

Supplement: Additional file 4 — Corroboration of the microarray gene expression data by RT-qPCR on Li2 mutant and WT fibers. Gene expression profiles of twelve genes selected to verify the microarray gene expression data. Affymetrix probeset IDs and predicted gene products are shown on the graph titles for each gene. The DPA time-points that revealed a significant (≥ 2-fold; p-value < 0.05) difference in transcript abundance are indicated by an asterisk and the fold-change in transcript abundance is shown on the graphs above each indicated time-point. Error bars indicate standard deviation from 3 biological replicates. [file 1471-2164-12-445-S4.TIFF]
